# Renal Biomarkers as Predictors of Renal Damage in Patients With Type 2 Diabetes

**DOI:** 10.1155/bmri/4352812

**Published:** 2026-06-02

**Authors:** Mohamed Osman Ali, DafaAllah Hassan Billal, GadAllah Modawe, Abdelhakam G. Tamomh, Ahmed Ibn Edriss

**Affiliations:** ^1^ Faculty of Medical Laboratory Sciences, University of El Imam El Mahdi, Kosti, Sudan, mahdi.edu.sd; ^2^ Faculty of Medicine and Health Sciences, Omdurman Islamic University, Omdurman, Sudan, oiu.edu.sd

**Keywords:** creatinine, diabetes mellitus, renal function, Sudan, urea

## Abstract

**Purpose:**

Diabetes mellitus (DM) is a chronic metabolic disease of global health concern. Type 2 diabetes mellitus (T2DM) is characterized by varying degrees of insulin resistance and impaired insulin secretion and can lead to renal dysfunction. This study, therefore, evaluated the prognostic potential of renal biomarkers as predictors of kidney damage in Sudanese T2DM.

**Materials and Methods:**

An analytical case–control hospital‐based study was conducted among patients attending the Kosti Diabetic Center (KDC) from March to October 2023. A total of 400 individuals were enrolled in the study: 200 with T2DM and 200 without T2DM as the control group. Their age and sex were matched. Serum urea, fasting blood glucose (FBG), creatinine, and albumin levels were estimated using a semiautomated biochemical analyzer; HbA1c levels were determined with a Getein 1100 analyzer. Data were analyzed using SPSS, and descriptive, *t*‐tests, and multivariate analyses were performed.

**Results:**

Plasma urea (95% CI = 11.12 − 17.02, *p* < 0.001), creatinine (95% CI = 0.442 − 0.606, *p* < 001), urine albumin (95% CI = 111.78 − 138.63, *p* < 0.001), and albumin‐to‐creatinine ratio (ACR) (95% CI = 20.03 − 28.92, *p* < 0.001) were significantly higher in the case group than in the control group, while serum albumin (95% CI = −1.73 to −1.46, *p* < 0.001) and urine creatinine (95% CI = −103.79 to −78.02, *p* < 0.001) levels were significantly lower in the case group than in the control group. The results showed no significant differences in biochemical parameters by sex (*p* > 0.05). Serum urea and creatinine levels were significantly higher in patients with poor glycemic control (HbA1c ≥ 8) than in those with good glycemic control (HbA1c < 8) (*p* = 0.002 and *p* < 0.001, respectively). ACR weakly correlated with HbA1c and FBG levels (Spearman′s correlation coefficient, *r* = 0.229, *p* < 0.001, and *r* = 0.201, *p* < 0.001, respectively) but strongly correlated with the duration of diabetes. There was a significant positive correlation between ACR and serum creatinine (*r* = 0.261, *p* < 0.001). At the same time, there was a significant negative correlation between ACR and serum albumin (*r* = −0.381, *p* < 0.001) and serum albumin and serum creatinine (*r* = −0.590, *p* < 0.001).

**Conclusion:**

Patients with Type 2 diabetes have significantly higher serum urea, creatinine, and ACR levels. Renal dysfunction in Type 2 diabetics is associated with poor glycemic control and the duration of the disease.

## 1. Introduction

Diabetes mellitus (DM) is a chronic metabolic disease characterized by persistent hyperglycemia due to defects in insulin secretion, insulin action, or both [1]. DM is a global health concern, with the International Diabetes Federation estimating that over 500 million individuals worldwide are affected [1–3]. The illness imposes a significant burden on public healthcare systems due to its long‐term complications, which include cardiovascular disease, retinopathy, neuropathy, and chronic kidney disease (CKD) [4–6]. The two main forms of DM are Type 1 diabetes mellitus (T1DM) and Type 2 diabetes mellitus (T2DM) [5]. About 90% of people with diabetes are T2DM, with associated age‐adjusted mortality approximately twice that of the nondiabetics, coupled with a 5–10‐year reduction in life expectancy [6, 7].

Among the major complications of DM, CKD stands out due to its high prevalence and severe health consequences [8]. Diabetic kidney disease (DKD), a leading cause of end‐stage renal disease (ESRD), results from the progressive damage to the glomeruli, tubules, and interstitium of the kidneys [9]. The interplay among hyperglycemia, hypertension, and inflammation accelerates renal damage [8–12]. Early identification and intervention are crucial, as CKD not only impairs renal function but also significantly increases the danger of cardiovascular anomalies and diabetes‐related deaths [11–14].

In early detection and prognosis of DKD, the key biochemical marker is albuminuria, defined as the abnormal excretion of albumin in the urine [15–17]. Microalbuminuria can serve as a predictor of renal failure and cardiovascular risk [14–16]. Albuminuria reflects glomerular and endothelial dysfunction and is clinically used to stratify risk and guide drug interventions. Monitoring and evaluating albuminuria allow for adjustments to treatment, such as the use of renin–angiotensin system inhibitors, which can slow CKD progression and improve outcomes in patients with diabetes [17–20]. In patients with diabetes, monitoring kidney function is essential for early detection and prevention of CKD. Serum creatinine is a routinely used biochemical marker for estimating the glomerular filtration rate (eGFR), which reflects overall kidney function. However, serum creatinine parameter levels can remain within normal range even in the presence of early kidney damage, making it a relatively late indicator [21–23]. To improve early detection of kidney damage, the urinary albumin‐to‐creatinine ratio (ACR) has become a key tool for assessing albuminuria, one of the earliest indicators of DKD. ACR can detect both microalbuminuria and macroalbuminuria, indicating progressive glomerular dysfunction [24–26]. Unlike serum creatinine alone, ACR provides insight into the structural integrity of the glomerular filtration barrier and communicates both onset and progress [26, 27]. Together, serum creatinine and urinary ACR offer a complementary and more comprehensive assessment of renal health in individuals with DM, providing an avenue for earlier detection and intervention.

Despite the reported global burden of DKD, there exist diagnostic challenges in resource‐limited regions across the globe, and the Kosti area of Sudan is no exception. While serum creatinine is the primary biomarker used in local clinical settings to monitor renal health, it is an insensitive biomarker for early‐stage detection, often remaining within normal ranges until more than half of kidney function is lost. In the White Nile State of Sudan, there is limited information on the prevalence of microalbuminuria, particularly among T2DM patients. Without localized data on the ACR, healthcare providers in Kosti lack the empirical evidence needed to implement early screening protocols, which could prevent irreversible renal failure and decrease the high burden of cardiovascular mortality. Therefore, this study is aimed at evaluating renal function parameters among Sudanese with T2DM in Kosti City, thereby providing essential data for early detection and improved clinical management in the area.

## 2. Materials and Methods

### 2.1. Study Design, Area, and Population

This analytical case–control hospital‐based study was conducted at the Clinical Laboratory Unit of the Kosti Diabetic Center (KDC), White Nile State, Sudan. The study covered the period from March to October 2023. KDC is the only diabetic healthcare facility in Kosti City [28]. A total of 400 participants were enrolled in the study, comprising 200 T2DM cases and 200 apparently healthy, age‐ and sex‐matched controls. The T2DM cases were subdivided into two groups: 90 poor glycemic patients (HbA1c ≥ 8*%*) and 110 good glycemic control patients (HbA1c < 8*%*).

### 2.2. Inclusion and Exclusion Criteria

The study targeted patients aged 40–70 years with long‐term (7–10 years) T2DM, with normal blood pressure and body mass index (BMI), and seeking care at the KDC. Patients in this category who had fasted for 8–12 h at the time of visit to the KDC were included in the study. On the contrary, the study excluded patients with renal disease, liver disease, autoimmune disease, and HIV, those receiving chemotherapy and radiotherapy, those with hormonal disturbances, pregnant women, smokers (participants with any current or past tobacco use verified by carbon monoxide or cotinine levels), and alcoholics (using the criteria for alcohol use disorder [AUD], e.g., a DSM‐5 diagnosis or exceeding National Institute on Alcohol Abuse and Alcoholism [NIAAA] heavy drinking thresholds [e.g., > 14 drinks/week for men or > 7 for women]). The apparently healthy controls were nondiabetic individuals aged 40–70 years who exhibited normal blood pressure and BMI, irrespective of sex.

### 2.3. Data Collection and Sample Size

Primary data were collected using a structured questionnaire adapted from available literature. After explaining the study to the participants, those who volunteered to participate completed the questionnaire. The questionnaire captured participants′ age and sex. Secondary data were collected from laboratory results as follows: blood pressure (measured twice on the day of data collection using a validated, automatic, cuff‐style upper‐arm blood pressure monitor) and BMI (calculated using the formula: weight in kg/height in m^2^). Biochemical analyses were performed on blood specimens collected from the participants to measure fasting blood glucose, plasma urea, creatinine, albumin, and HbA1c, while their urine was analyzed for urine creatinine and albumin levels.

### 2.4. Sampling and Processing

Following consent and adhering to the patient charter, 5 mL of venous blood was aseptically collected: 2.5 mL was placed into an EDTA container and then tested for HbA1c (mmol/mol), while the remaining 2.5 mL was placed into a lithium heparin blood container and centrifuged for 5 min at 3000 rpm, and the plasma was analyzed immediately for glucose, urea, creatinine, and albumin levels. In addition, 10 mL of midstream urine samples were collected in a clean, dry urine container and tested for albumin (mg/L) and creatinine (mmol/L). Serum urea (mmol/L), FBG (mmol/L), creatinine (*μ*mol/L), and albumin (g/L) levels were estimated using spectrophotometric methods (spectrophotometer, semiautomated Mindray BA‐88A, Shenzhen Mindray Bio‐Medical Electronics Co., Ltd, China). HbA1c levels were measured using a Getein 1100 analyzer (Biotech, Inc., China). The ACR (mg/mmol) was calculated by dividing the urine albumin level by the urine creatinine (ACR = urine albumin/urine creatinine).

### 2.5. Ethical Considerations

The Faculty of Medical Laboratory Sciences Ethics Committee at the University of El Imam El Mahdi approved the study (approval code: EA.FMLS.UEE.2022‐6). Site permission was obtained from the management of the KDC. Written informed consent was obtained from all participants in the study. This study was conducted in compliance with the Helsinki Declaration.

### 2.6. Data Analysis

Renal biomarkers and sociodemographic data were described in terms of mean values because the data were normally distributed. The independent *t*‐test was used to compare the differences in renal biomarker values between T2DM patients and healthy individuals. Multivariate analysis was applied to compare the independent variables (age, gender, BMI, and duration of DM) in T2DM patients and healthy individuals. The strength of the relationships between ACR, HbA1c, FBG, and diabetes duration among diabetic patients and between ACR, serum albumin, and serum creatinine across the two groups was assessed using the Spearman correlation test. The analyses were performed using SPSS Version 25.0; outcomes were considered statistically significant when *p* < 0.05.

## 3. Results

The participants′ demographics revealed a mean age of 59.3 ± 11.6. Specifically, the case groups had a mean age of 61.5 ± 11.9, while the control group had a mean age of 56.6 ± 10.6. Gender‐wise, 180 (45%) were female, and 220 (55%) were male. The duration of T2DM averaged 4–7 years. The mean ± SD BMI, FBG, and HbA1c (%), in the case group, were 24.5 ± 3.6, 132.5 ± 36.2, and 7.3 ± 2.00, respectively. There was no significant difference between age and BMI nor between the two groups (*p* = 0.063 and *p* = 0.206, respectively).

**Figure 1:**
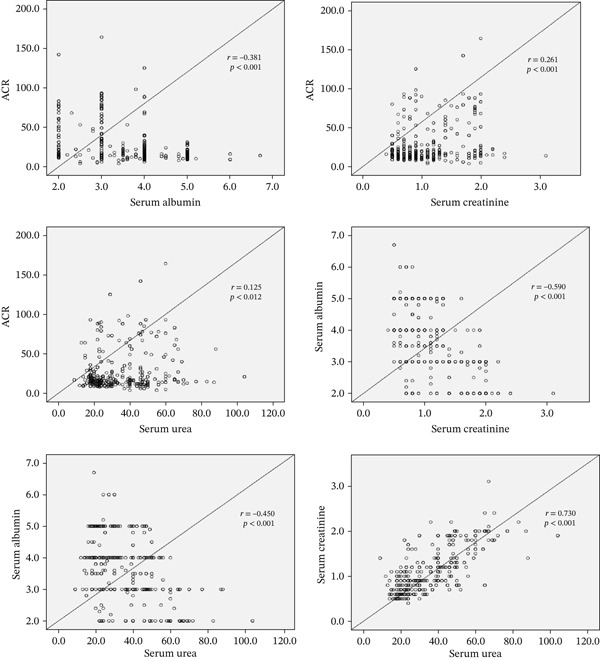
Relationship between ACR (mg/mmol), serum albumin (mg/L), serum creatinine (*μ*mol/L), and serum urea (mmol/L) among the two groups.

In Table 1, the mean ± SD of plasma urea, creatinine, albumin, urine creatinine, urine albumin, eGFR, and ACR in the case group was 41.8 ± 17.6, 1.3 ± 0.5, 2.9 ± 0.6, 64.5 ± 78.6, 144.6 ± 98.6, 65.7 ± 30, and 38.0 ± 31.0, respectively, while that in the control group was 27.0 ± 10.7, 0.8 ± 0.2, 4.4 ± 0.6, 153.4 ± 52.6, 21.8 ± 4.5, 80.04 ± 32, and 15.7 ± 5.0, respectively. The mean level of urea in the case group (41.8 mmol/L) is a critical, life‐threatening level of uremia often associated with ESRD or acute kidney injury. A creatinine level of 1.3 mg/dL is abnormally high and indicates a significant decrease in kidney filtration capacity. In contrast, the control group′s level is within the normal range, indicating that the kidneys are efficiently clearing metabolic waste from the blood. A level of 2.9 ± 0.6 g/L represents severe hypoalbuminemia, a critical finding in diabetic patients that often results from massive proteinuria; conversely, the level in the control group indicates healthy liver function and adequate protein retention by the kidneys. The elevation in the ACR and albuminuria among diabetic patients is a hallmark of early diabetic nephropathy, which significantly increases the future risk of progressive renal decline and cardiovascular events. An eGFR of 80.04 ± 32 mL/min/1.73 m^2^ is considered normal for healthy adults, particularly as kidney capacity naturally declines with age. In contrast, an eGFR of 65 mL/min/1.73 m^2^ indicates Stage 2 CKD, indicating an 18% loss in filtration efficiency compared to the control group. The serum urea (95% CI = 11.12 − 17.02, *p* < 0.001), creatinine (95% CI = 0.442 − 0.606, *p* < 0.001), urine albumin (95% CI = 111.78 − 138.63, *p* < 0.001), eGFR (95% CI = −20.44 to −8.07, *p* < 0.001), and ACR (95% CI = 20.03 − 28.92, *p* < 0.001) were significantly higher in the case group than in the control group (*p* < 0.001), whereas the serum albumin (95% CI = −1.73 to −1.46, *p* < 0.001) and urine creatinine (95% CI = −103.79 to −78.02, *p* < 0.001) levels were significantly lower in the case group than in the control group (*p* < 0.001). There were insignificant differences in biochemical parameters between male and female diabetic patients (*p* > 0.05), as shown in Table 2. Diabetic patients with uncontrolled hyperglycemia (HbA1c ≥ 8) had significantly higher plasma urea, creatinine, and albumin (*p* = 0.002,*p* < 0.001, and*p* < 0.001, respectively) (see Table 3). ACR weakly correlated with HbA1c and FBG levels (*r* = 0.229, *p* < 0.001; *r* = 0.201, *p* < 0.001) but strongly correlated with the duration of diabetes (*r* = 0.554, *p* < 0.001) as explained in Table 4. We further assessed the association between eGFR, ACR, serum creatinine, serum albumin, and serum urea with the independent variables (age, gender, BMI, and duration of DM) for renal dysfunction using multivariate analysis (Table 5). There was a significant positive correlation between ACR and serum creatinine (*r* = 0.261, *p* < 0.001). At the same time, there was significant negative correlation between ACR and serum albumin (*r* = −0.381, *p* < 0.001), a weak positive correlation between ACR and serum urea (*r* = 0.125, *p* = 0.012), a high negative correlation between serum albumin and serum creatinine (*r* = −0.590, *p* < 0.001) and also between serum albumin and serum urea (*r* = −0.450, *p* < 0.001), and additionally a high positive correlation between serum creatinine and serum urea (*r* = 0.730, *p* < 0.001) (Table 6 and Figure 1).

**Table 1:** Differences in biochemical parameters between the two groups.

Parameters	Case group (*n* = 200)	Control group (*n* = 200)	95% CI	*p* value
Serum urea (mmol/L)	41.8 ± 17.6	27.0 ± 10.7	(11.12–17.02)	< 0.001
Serum creatinine (*μ*mol/L)	1.3 ± 0.5	0.8 ± 0.2	(0.442–0.606)	< 0.001
Serum albumin (g/L)	2.9 ± 0.6	4.4 ± 0.6	(−1.73 to −1.46)	< 0.001
Urine creatinine (mmol/L)	64.5 ± 78.6	153.4 ± 52.6	(−103.79 to −78.02)	< 0.001
Urine albumin (mg/L)	144.6 ± 98.6	21.8 ± 4.5	(111.78–138.63)	< 0.001
ACR (mg/mmol)	38.0 ± 31.0	15.7 ± 5.0	(20.03–28.92)	< 0.001
eGFR (mL/min/1.73 m^2^)	65.7 ± 30	80.04 ± 32	(−20.44 to −8.07)	< 0.001

**Table 2:** Differences in biochemical parameters by gender among diabetic patients attending the Kosti Diabetic Center, Kosti City, White Nile State, Sudan.

Parameter	Female (*n* = 90)	Male (*n* = 110)	*p* value
Serum urea (mmol/L)	39.1 ± 17.3	45.3 ± 17.5	0.222
Serum creatinine (*μ*mol/L)	1.2 ± 0.5	1.3 ± 0.5	0.293
Urine creatinine (mmol/L)	68.3 ± 80.9	59.4 ± 75.8	0.392
Urine albumin (mg/L)	137.2 ± 91.0	154.2 ± 107.8	0.183
ACR (mg/mmol)	40.2 ± 31.1	35.1 ± 31.0	0.501

**Table 3:** Differences in renal biomarkers according to good and poor glycemic control in patients with Type 2 diabetes attending the Kosti Diabetic Center, Kosti City, White Nile State, Sudan.

Parameters	*H* *b* *A*1*c* < 8 (*n* = 110)	*H* *b* *A*1*c* ≥ 8 (*n* = 90)	*p* value
Serum urea (mmol/L)	38.7 ± 17.0	47.4 ± 17.3	0.002
Serum creatinine (*μ*mol/L)	1.2 ± 0.5	1.5 ± 0.4	< 0.001
Serum albumin (g/L)	3.06 ± 0.66	2.59 ± 0.60	< 0.001
ACR (mg/mmol)	39.06 ± 33.6	41.82 ± 28.5	0.546

**Table 4:** Correlation of ACR with HbA1c, FBG, and duration of diabetes among diabetic patients attending the Kosti Diabetic Center, Kosti City, White Nile State, Sudan.

Parameters	*R*	*p* value
ACR (mg/mmol) vs. HbA1c (mmol/mol)	0.229	< 0.001
ACR (mg/mmol) vs. FBG (mmol/L)	0.201	< 0.001
ACR (mg/mmol) vs. duration of diabetes (years)	0.554	< 0.001

**Table 5:** Multivariate analysis to assess the association of eGFR, ACR, serum creatinine, serum albumin, and serum urea with the independent variables for renal dysfunction among diabetic patients.

Independent variables	Serum urea F (*p* value)	Serum creatinine F (*p* value)	Serum albumin F (*p* value)	ACR F (*p* value)	eGFR F (*p* value)
Age	9.7 (< 0.001)	10.3 (< 0.001)	34.5 (< 0.001)	120.08 (< 0.001)	0.43 (0.995)
Gender	2.37 (0.129)	1.96 (0.167)	0.002 (0.964)	9.74 (0.003)	4.28 (0.043)
BMI	7.01 (< 0.001)	10.96 (< 0.001)	26.42 (< 0.001)	222.26 (< 0.001)	0.474 (0.944)
Duration of DM	10.02 (< 0.001)	15.96 (< 0.001)	19.03 (< 0.001)	711.11 (< 0.001)	0.064 (0.979)

**Table 6:** Relationship between different renal biomarkers among the two groups.

Parameters	Case group		Control group	
	*r*	*p* value	*r*	*p* value
ACR (mg/mmol) vs. serum albumin (mg/L)	−0.21	0.766	−0.086	0.228
ACR (mg/mmol) vs. serum creatinine (*μ*mol/L)	0.012	0.871	−0.011	0.880
ACR (mg/mmol) vs. serum urea (mmol/L)	−0.051	0.473	−0.041	0.568
Serum albumin (mg/L) vs. serum creatinine (*μ*mol/L)	−0.438	< 0.001	−0.154	0.029
Serum albumin (mg/L) vs. serum urea (mmol/L)	−0.381	< 0.001	−0.112	0.114
Serum creatinine (*μ*mol/L) vs. serum urea (mmol/L)	0.694	< 0.001	0.590	< 0.001

## 4. Discussion

The goal of this study was to evaluate renal biomarkers as predictors of kidney damage among Sudanese patients with T2DM in Kosti, White Nile State, Sudan. Our results showed a significant increase in FBG, HbA1c, plasma urea, and plasma creatinine levels in T2DM cases compared with healthy controls. These findings suggest that sustained elevation of these markers predisposes to renal damage, as previously reported [29]. As previously reported, Faheem et al. found a significant increase in BFG, HbA1c, serum creatinine, and urea levels in patients with T2DM compared to normal controls [30]. Singh et al. also noted that blood urea, serum creatinine, and FBG were significantly higher in T2DM than in nondiabetics [31]. Furthermore, Kundu et al. reported elevated levels of FBG, HbA1c, and serum urea in T2DM, similar to observations by Shrestha et al. [32] and Seçmeler et al. [33]. Collectively, these observations point to a persistent risk of renal damage, especially when glycemic levels are poorly controlled.

Hyperglycemia is a risk factor for diabetic nephropathy and is associated with glomerular changes, including mesangial matrix expansion, sclerosis, basal membrane thickening, tubular epithelium flattening, and lumen widening [34, 35]. There is a spectrum of changes in CKD, with well‐defined functional progression from hyperfiltration to micro‐ or macroalbuminuria to renal dysfunction. This result was supported by Streja et al., who reported that ACR was significantly higher in patients with diabetes than in controls [29, 36].

In our study, there were insignificant differences in biochemical parameters between males and females with diabetes; however, males showed slightly higher creatinine levels, which may be due to their higher muscle mass. This result is in line with the findings of Shrestha et al. and Ashavaid et al., who reported that sex‐wise variation affected serum creatinine levels only [32, 35].

Additionally, our study revealed significant increases in plasma urea and creatinine levels in patients with poor glycemic control compared with those with good glycemic control. HbA1c has a special affinity for oxygen, thereby causing tissue anoxia, and plays a role in the pathogenesis of micro‐ and macroangiopathy.

In Type 2 diabetic patients, the duration of diabetes was the strongest predictor and could elevate glycemic control (HbA1c) and predict increased microalbumin excretion rate [31]. Our present results confirm what Faheem et al. observed: a significant increase in serum creatinine and urea levels in diabetes with poor glycemic control compared with those with good glycemic control [30]. Shrestha et al. noted that blood urea and serum creatinine were significantly increased in poorly controlled diabetics compared with well‐controlled diabetics, and Haque et al. reported a significant increase in serum creatinine level in diabetic patients with poor glycemic control compared with those with good glycemic control and normal controls [29, 30, 32].

Measurement of HbA1c is used in long‐term monitoring of DM and to assess whether there is a link between early glomerular changes and uncontrolled hyperglycemia. The study revealed that ACR significantly and positively correlates with FBG, HbA1c, and the duration of DM. This suggested that the duration of DM and uncontrolled hyperglycemia were strong predictors of developing microalbuminuria. This result is supported by Awadalla et al., who have reported a significant positive correlation between ACR, FBG, and HbA1c. Haque et al. also observed a significant correlation between HbA1c and ACR, while Sheikh et al. and Agarwal et al. reported a strong correlation between renal dysfunction and duration of diabetes [31, 37–42].

In the current study, we further assessed, using multivariate analysis, the association of ACR, serum creatinine, serum albumin, and serum urea with the independent variables (age, gender, BMI, and duration of DM) for renal dysfunction. Our findings revealed that eGFR, ACR, and serum urea are intricately linked to demographic and clinical factors, with age and diabetes duration as the strongest independent predictors of decline. While eGFR decreases with age due to senescence, a prolonged duration of diabetes accelerates this process through chronic hyperglycemia, which elevates ACR and eventually reduces filtration capacity. Furthermore, gender influences these markers due to variations in muscle mass, which affect serum creatinine levels, while a high BMI is strongly associated with hyperfiltration and increased ACR.

In our study, a regression analysis revealed a significant positive correlation between ACR and serum creatinine, a significant negative correlation between ACR and serum albumin, and a weak positive correlation between ACR and serum urea. There was a high positive correlation between serum creatinine and serum urea, but there was a high negative correlation between serum creatinine and serum albumin. A strong negative association was found between serum albumin and serum urea. A high increase in ACR might indicate that the kidney is less effective at filtering waste products; therefore, it is an early sign of kidney damage and glomerular filtration rate reduction. These correlations strongly suggest kidney damage among Type 2 diabetic patients; therefore, the renal biomarkers assessed in our study can serve as indicators for diagnosing, staging, and monitoring kidney damage.

## 5. Conclusion

Diabetic patients had significantly higher serum urea, creatinine, and ACR levels. Patients with poorly controlled T2DM were more prone to renal damage, and ACR strongly correlates with the duration of DM. Therefore, serum urea, creatinine, and the ACR may serve as useful prognostic markers and predictors of renal damage, and good control of the blood glucose levels is required to prevent progressive renal impairment.

## Author Contributions

M.O.A., D.H.B., G.M., A.G.T., and A.I.E. conceived and designed the study. M.O.A. and D.H.B. implemented the study. A.G.T. supervised the study. A.G.T. conducted data analysis. M.O.A., D.H.B., G.M., A.G.T., and A.I.E. interpreted study results. M.O.A. wrote the first draft of the manuscript, while D.H.B., G.M., A.G.T., and A.I.E. reviewed and corrected the manuscript.

## Funding

No funding was received for this manuscript.

## Disclosure

All authors read and approved the final manuscript.

## Consent

The authors have nothing to report.

## Conflicts of Interest

The authors declare no conflicts of interest.

## Data Availability

The data that support the findings of this study are available on request from the corresponding author. The data are not publicly available due to privacy or ethical restrictions.
